# Supplementary material for: N-acetylneuraminic acid links immune exhaustion and accelerated memory deficit in diet-induced obese Alzheimer’s disease mouse model
Source: Nat Commun. 2023 Mar 9;14:1293. doi: 10.1038/s41467-023-36759-8 (PMC9998639; doi:10.1038/s41467-023-36759-8)
Supplement: Supplementary file 1 — Supplementary Information [file 41467_2023_36759_MOESM1_ESM.pdf]

Supplementary Information

N-acetylneuraminic acid links immune exhaustion and accelerated memory deficit in diet-induced obese Alzheimer's disease mouse model

Stefano Suzzi[1,#,*], Tommaso Croese[1,#], Adi Ravid[2], Or Gold[2], Abbe R. Clark[3], Sedi Medina[1], Daniel Kitsberg[2], Miriam Adam[2], Katherine A. Vernon[3], Eva Kohnert[3], Inbar Shapira[2], Sergey Malitsky[4], Maxim Itkin[4], Alexander Brandis[4], Tevie Mehlman[4], Tomer M. Salame[4], Sarah P. Colaiuta[1], Liora Cahalon[1], Michal Slyper[3], Anna Greka[3,$,*], Naomi Habib[2,$,*], and Michal Schwartz[1,$,*]

[1]Weizmann Institute of Science, Department of Brain Sciences, Rehovot, Israel

[2]The Hebrew University of Jerusalem, Edmond & Lily Safra Center for Brain Sciences, Jerusalem, Israel

[3]Broad Institute of MIT and Harvard, Cambridge, MA, USA; Department of Medicine, Brigham and Women's Hospital and Harvard Medical School, Boston, MA, USA

[4]Weizmann Institute of Science, Life Sciences Core Facilities, Rehovot, Israel

[#]These authors contributed equally

[$]These authors jointly supervised this work

*Correspondence: michal.schwartz@weizmann.ac.il (M.Schwartz); naomi.habib@mail.huji.ac.il (N.H.); agreka@broadinstitute.org (A.G.); stefano.suzzi@weizmann.ac.il (S.S.).

This file includes:

Supplementary Figures 1–16 and their legends

Supplementary Tables 1 and 2 and their legends

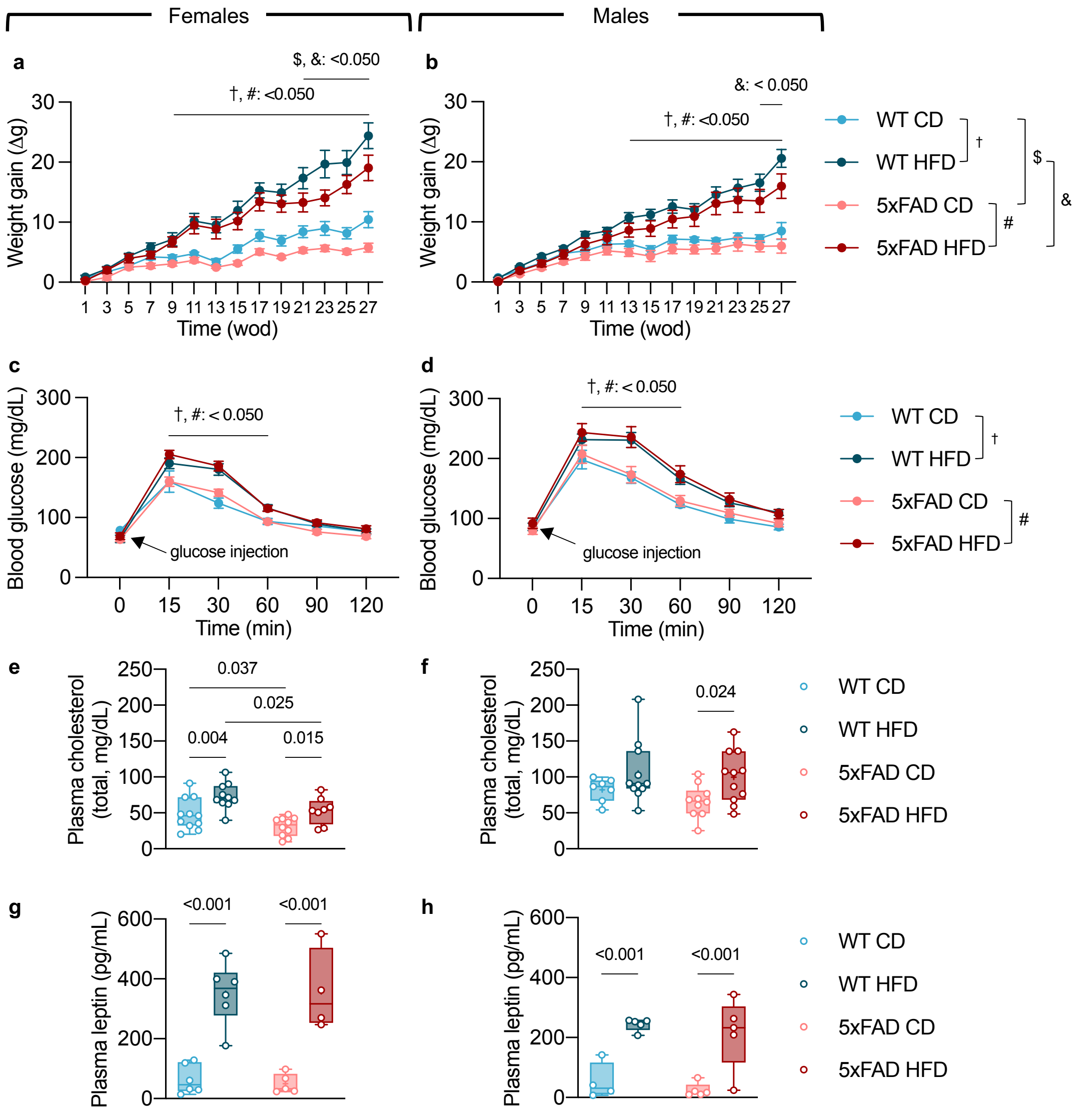

Supplementary Fig. 1: HFD-related metabolic changes in mice. a, **b**, Weight gain over time (weeks of diet, wod), means ± s.e.m. **c**, **d**, Glucose tolerance test at 13 wod, means ± s.e.m. **a–d**, Contrasts: †, WT CD vs. WT HFD; #, 5xFAD CD vs. 5xFAD HFD; $, WT CD vs. 5xFAD CD; &, WT HFD vs. 5xFAD HFD. Exact *P* values for each time point are reported in the Source Data file. **e**, **f**, Total plasma cholesterol levels. **g**, **h**, Total plasma leptin levels. **a**, **c**, **e**, **g**, Data from female mice. **b**, **d**, **f**, **h**, Data from male mice. Mice from two independent experiments, sample *n*: **a**, WT CD=9, WT HFD=10, 5xFAD CD=9, 5xFAD HFD=8; **b**, WT CD=7, WT HFD=12, 5xFAD CD=10, 5xFAD HFD=11; **c**, WT CD=11, WT HFD=10, 5xFAD CD=10, 5xFAD HFD=9; **d**, WT CD=8, WT HFD=12, 5xFAD CD=10, 5xFAD HFD=13; **e**, WT CD=11, WT HFD=10, 5xFAD CD=10, 5xFAD HFD=8; **f**, WT CD=7, WT HFD=11, 5xFAD CD=10, 5xFAD HFD=11; **g**, WT

CD=6, WT HFD=6, 5xFAD CD=5, 5xFAD HFD=4; **h**, WT CD=4, WT HFD=5, 5xFAD CD=5, 5xFAD HFD=5. Some of these animals were used for the analyses described in Fig. 1–4 and Supplementary Fig. 2–10. Statistical analyses: **a–d**, two-way within-subjects ANOVA followed by Fisher's LSD *post hoc* test; **e–h**, two-way ANOVA followed by Fisher's LSD *post hoc* test. **e–h**, Box plots represent the minimum and maximum values (whiskers), the first and third quartiles (box boundaries), the median (box internal line), and the mean (cross). Source data are provided as a Source Data file.

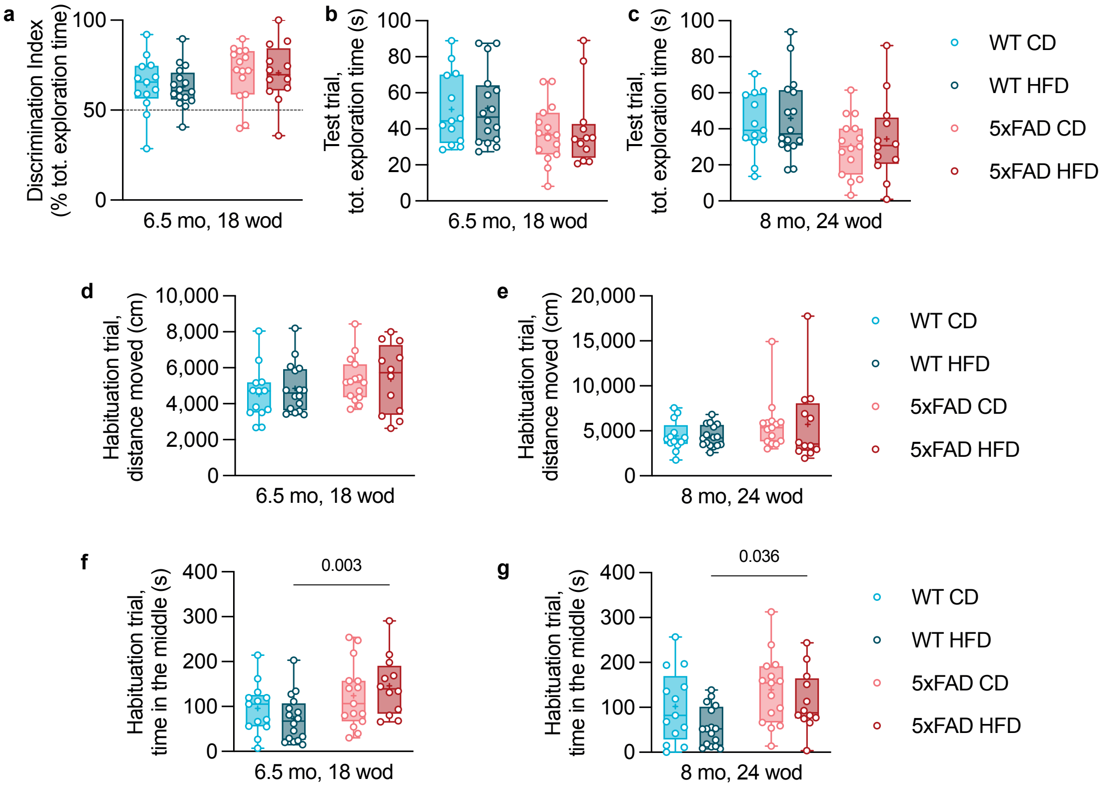

Supplementary Fig. 2: Effects of AD and HFD on novelty discrimination at 6.5 mo and locomotor activity/anxiety at 6.5 and 8 mo in mice. The mice are the same described in Fig. 1c, sample *n*: WT CD=13, WT HFD=16, 5xFAD CD=15, 5xFAD HFD=12. **a**, Novelty discrimination at 6.5 mo after 18 weeks of diet (wod). **b**, **c**, Total exploration time during the test trial of the NOR test at 6.5 (**b**) and 8 mo (**c**). **d**, **e**, Distance moved in the empty arena during the habituation trial of the NOR test at 6.5 (**d**) and 8 mo (**e**). **f**, **g**, Time spent in the middle of the empty arena during the habituation trial of the NOR test at 6.5 (**f**) and 8 mo (**g**). **a–g**, Statistical analyses: two-way ANOVA followed by Fisher's LSD *post hoc* test. Box plots represent the minimum and maximum values (whiskers), the first and third quartiles (box boundaries), the median (box internal line), and the mean (cross). Source data are provided as a Source Data file.

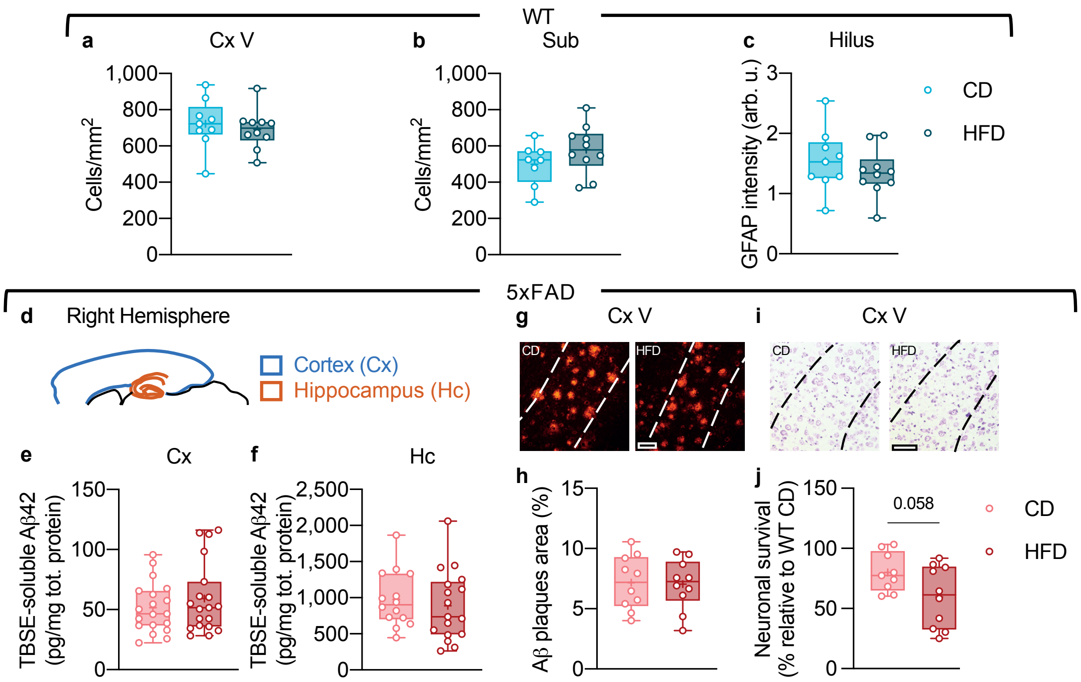

Supplementary Fig. 3: Effects of HFD on the mouse brain pathology. HFD did not affect neuronal density nor did it induce astrogliosis in WT mice after 28 weeks of diet. Assessment of neuronal density in the cortex layer V (Cx V; **a**) and subiculum (Sub; **b**) and expression of the astrocyte marker glial fibrillary acidic protein (GFAP) intensity in the hippocampal hilus (**c**) of WT mice. **c**, arb. u., arbitrary unit (average integrated density/100,000). Mice from two independent experiments, sample *n*: **a**, WT CD=9, WT HFD=10; **b**, WT CD=8, WT HFD=10; **c**, WT CD=9, WT HFD=10. **d–h,** HFD did not affect amyloid β load in 5xFAD mice. **d–f**, Determination of Tris Buffered Saline EDTA (TBSE)-soluble Aβ1-42 oligomers in the cortex (**d**, **e**) and hippocampus (**d**, **f**) of 5xFAD mice. **g**, **h**, Assessment of amyloid β plaques load. **i**, **j**, HFD marginally affected neuronal survival in the cortex of 5xFAD mice. **g**, **i**, Representative images, left: CD, right: HFD, scale bars: 70 μm. **e**, **f**, **h**, **j**, Mice from two independent experiments, sample *n*: **e**, 5xFAD CD=19, 5xFAD HFD=19; **f**, 5xFAD CD=14, 5xFAD HFD=17; **h**, same animals as in Fig. 1f, sample *n*: 5xFAD CD=10, 5xFAD HFD=10; **j**, same animals as in Fig. 1h, sample *n*: 5xFAD CD=9, 5xFAD HFD=10. **j**, Data normalized by average WT CD value (**a**). **a–c**, **e**, **f**, **h**, **j**, Statistical analyses: two-tailed unpaired Student's *t*-test. Box plots represent the minimum and maximum values (whiskers), the first and third quartiles (box boundaries), the median (box internal line), and the mean (cross). Source data are provided as a Source Data file.

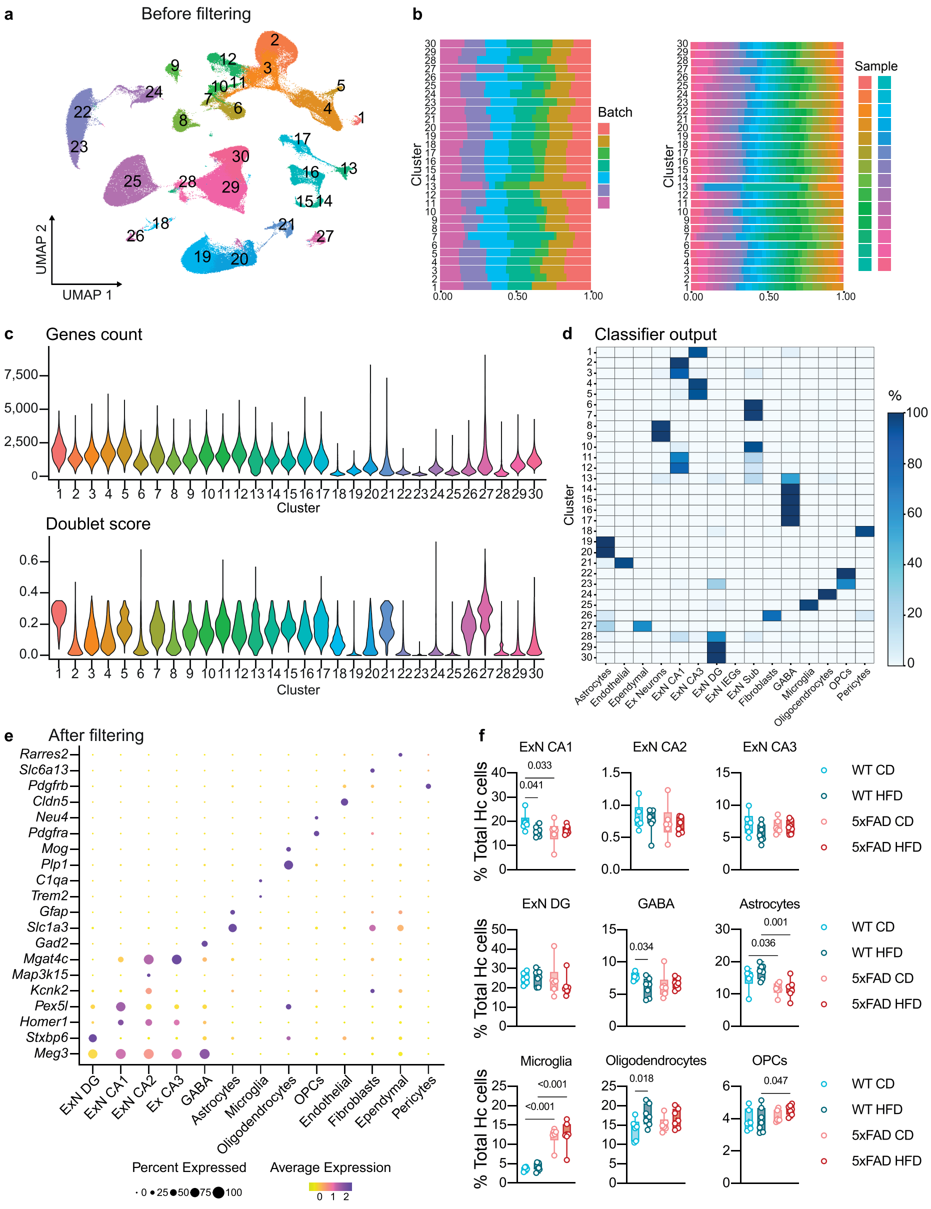

Supplementary Fig. 4. sNuc-Seq of the mouse hippocampus. a, UMAP embedding of pre-filtered 269,503 single nuclei profiles (sNuc-Seq), featuring doublet cells and clusters that were excluded from the analysis, colored by clusters. Mice are the same described in Fig. 2a–m, sample *n*=28. **b**, Similar distribution of cells per cluster across samples and batches. Cluster annotations are as in **a**. The percentage of cells per cluster, from the different batches (left) and from each sample (right), are colored by batch or sample. **c**, Violin plots showing the distribution of number of genes (top) and doublet score (bottom) detected in each cluster (Methods, *Quality controls for*

sequencing and pre-processing of sNuc-Seq data section). Cluster annotations are as in **a**. **d**, Output of cell type classifier (logistic regression; Methods, *Identification of clusters' cell types* section). Heatmap showing the fraction of cells classified to known hippocampal cell types (X axis) out of each cell cluster (Y axis). Cluster annotations are as in **a**. Abbreviations: Ex Neurons, excitatory neurons; ExN CA1–3, excitatory neurons of the *cornu Ammonis* regions 1–3; ExN DG, excitatory neurons of the dentate gyrus; ExN IEGs, recently activated excitatory neurons expressing immediate early genes; GABA, GABAergic neurons; OPCs, oligodendrocyte precursor cells; Sub, subiculum. **e**, **f**, Cell type annotations post filtering. **e**, Dot plot featuring the expression of marker genes (color scale) across cell types and the percentage of cells expressing them (dot size). **f**, Changes in the frequency of neuronal and non-neuronal cell types across experimental conditions. Hc, hippocampus. For quantifications and statistical analyses, 219,237 nuclei were included from *n*=26 samples: WT CD=6, WT HFD=7, 5xFAD CD=6, 5xFAD HFD=7 (Methods, *Cell fraction estimations and statistics* section). Statistical analyses: two-way ANOVA followed by Fisher's LSD *post hoc* test. Box plots represent the minimum and maximum values (whiskers), the first and third quartiles (box boundaries), the median (box internal line), and the mean (cross). Source data are provided as a Source Data file.

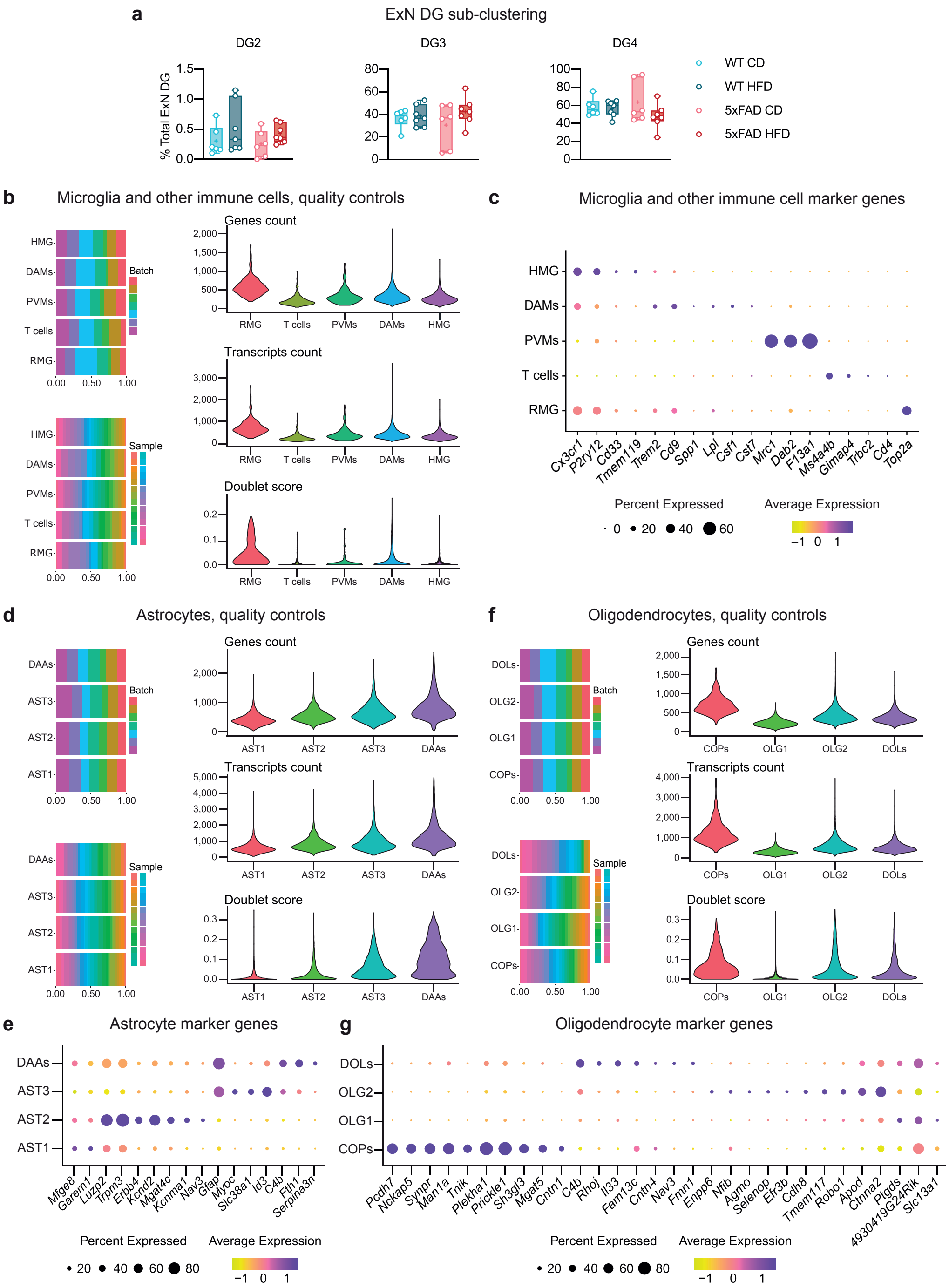

Supplementary Fig. 5: Sub-clustering of mouse DG neurons and glial cells. a, Changes in frequency of cell states of dentate gyrus granule neurons (ExN DG) across all experimental groups. Sub-clustering analysis identified four clusters (DG1–4), as shown in Fig. 2b; plot relative to DG1 is shown in Fig. 2c. Mice are the same described in Fig. 2a–m, sample *n*=26: WT CD=6, WT HFD=7, 5xFAD CD=6, 5xFAD HFD=7 (Methods, *Cell fraction estimations and statistics*

section). Statistical analyses: two-way ANOVA followed by Fisher's LSD *post hoc* test. Box plots represent the minimum and maximum values (whiskers), the first and third quartiles (box boundaries), the median (box internal line), and the mean (cross). Source data are provided as a Source Data file. **b**, Quality controls for microglia and other immune cells sub-clustering analysis (sample n=28). Abbreviations: DAMs, disease-associated microglia; HMG, homeostatic microglia; PVMs, perivascular macrophages; RMG, replicating microglia. Left: frequency of cells per cluster, from the different batches (top) and from each sample (bottom). Right: violin plots showing the distribution of number of genes (top), number of transcripts (middle), and doublet score (bottom) detected in each cluster. **c**, Dot plot featuring the expression of marker genes (color scale) and the percentage of cells expressing them (dot size) of microglia states and other immune cell types. **d–g**, Quality controls for astrocytes (**d**, **e**) and oligodendrocytes (**f**, **g**) sub-clustering analysis (sample n=28). Abbreviations: AST1–3, astrocyte clusters 1–3; COPs, committed oligodendrocyte precursors; DAAs, disease-associated astrocytes; DOLs, disease-associated oligodendrocytes; OLG1, 2, oligodendrocyte clusters 1, 2. Left: frequency of cells per cluster, from the different batches (top) and from each sample (bottom). Right: violin plots showing the distribution of number of genes (top), number of transcripts (middle), and doublet score (bottom) detected in each cluster. **e**, **g**, Dot plots featuring the expression of marker genes (color scale) and the percentage of cells expressing them (dot size) of astrocytes (**e**) and oligodendrocytes (**g**).

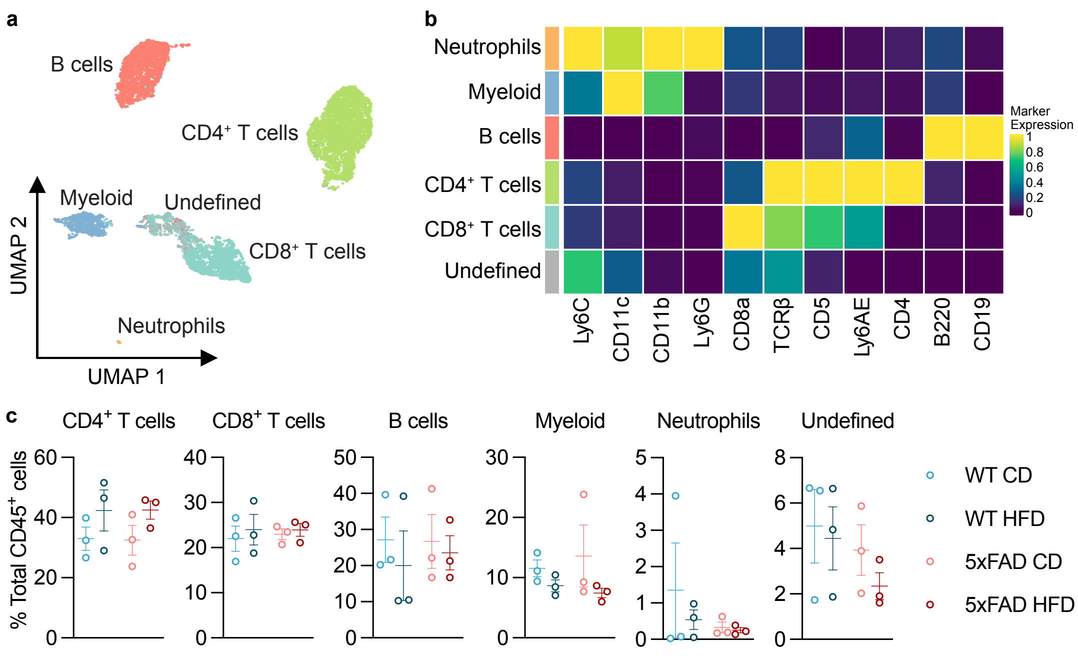

Supplementary Fig. 6: Effects of AD and HFD on the mouse blood immune profile. a, UMAP embedding of immune cell clusters in the blood (1,000 CD45$^+$ cells, randomly selected from each animal). FlowSOM-based immune cell populations are overlaid as a color dimension. **b**, Mean expression levels of markers detected across immune subpopulations. **c**, Frequency of the identified blood immune cell clusters. Data from one experiment, sample *n*: WT CD=3 (males), WT HFD=3 (2 females, 1 male), 5xFAD CD=3 (females), 5xFAD HFD=3 (females). Statistical analyses: two-way ANOVA followed by Fisher's LSD *post hoc* test. Bars show the mean and the standard error of the mean. Source data are provided as a Source Data file.

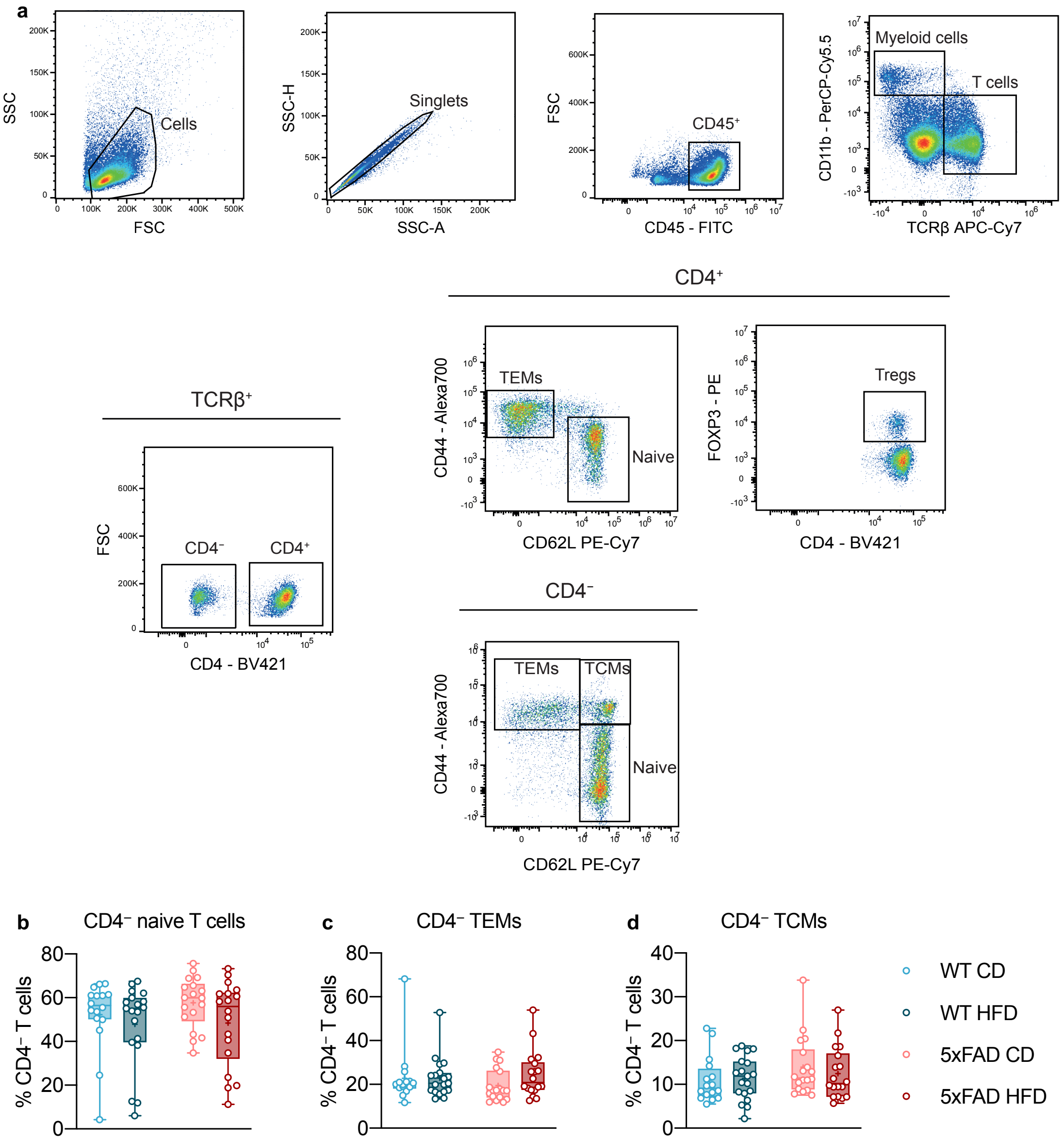

Supplementary Fig. 7: HFD did not affect the splenic $CD4^{-}$ T-cell compartment in mice. Mice are the same described in Fig. 3a–c, sample *n*: WT CD=16, WT HFD=19, 5xFAD CD=18, 5xFAD HFD=18. **a**, Flow cytometric characterization of the $CD4^{+}$ and $CD4^{-}$ T-cell compartments, gating strategy. **b–d**, Quantification of splenic frequencies of $CD4^{-}$ naive T cells ($CD44^{low}CD62L^{high}$; **b**), $CD4^{-}$ TEMs ($CD44^{high}CD62L^{low}$; **c**), and $CD4^{-}$ TCMs ($CD44^{high}CD62L^{high}$; **d**). Statistical analyses: two-way ANOVA followed by Fisher's LSD *post hoc* test. Box plots represent the minimum and maximum values (whiskers), the first and third quartiles (box boundaries), the median (box internal line), and the mean (cross). Source data are provided as a Source Data file.

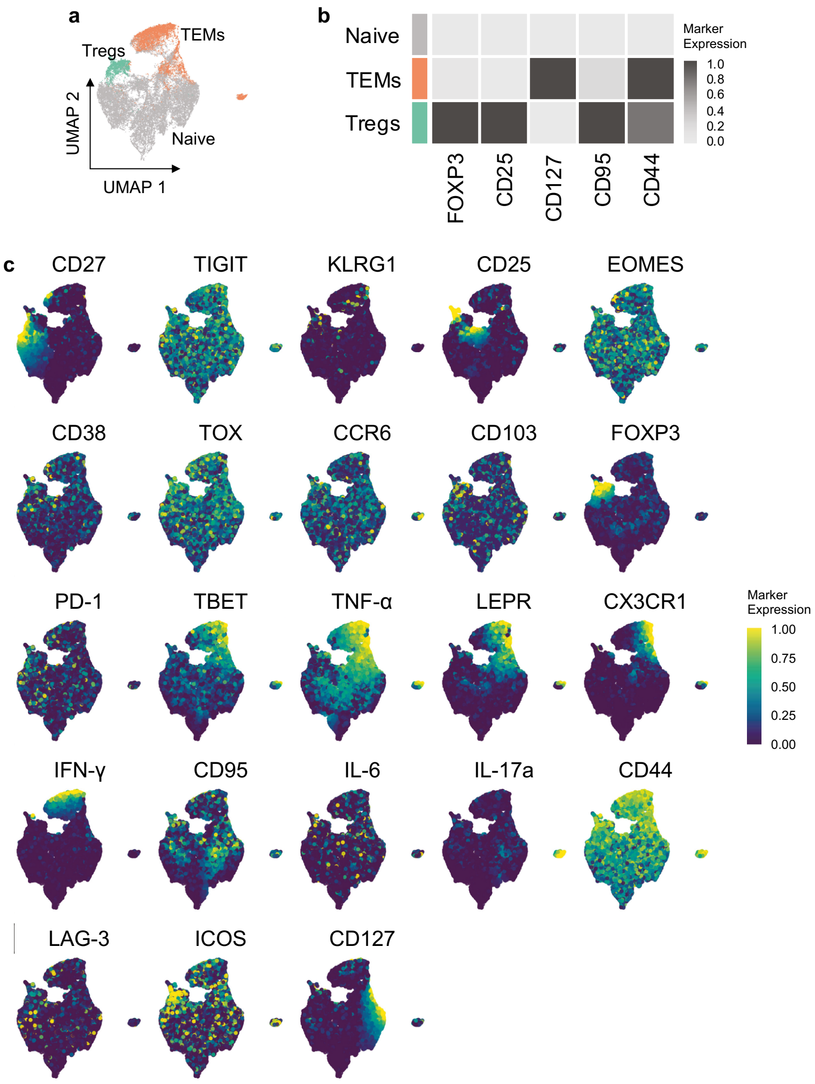

Supplementary Fig. 8: Single-cell profiling of mouse splenic CD4⁺ T cells by mass cytometry. Mice are the same described in Fig. 3e–f, sample *n*: WT CD=5, WT HFD=5, 5xFAD CD=5, 5xFAD HFD=5. **a**, UMAP embedding of CD4⁺ cell clusters (2,000 cells, randomly selected from each animal). FlowSOM-based immune populations are overlaid as a color dimension. **b**, Mean expression levels of the markers used for UMAP visualization and FlowSOM clustering. **c**, The expression of each indicated marker is overlaid.

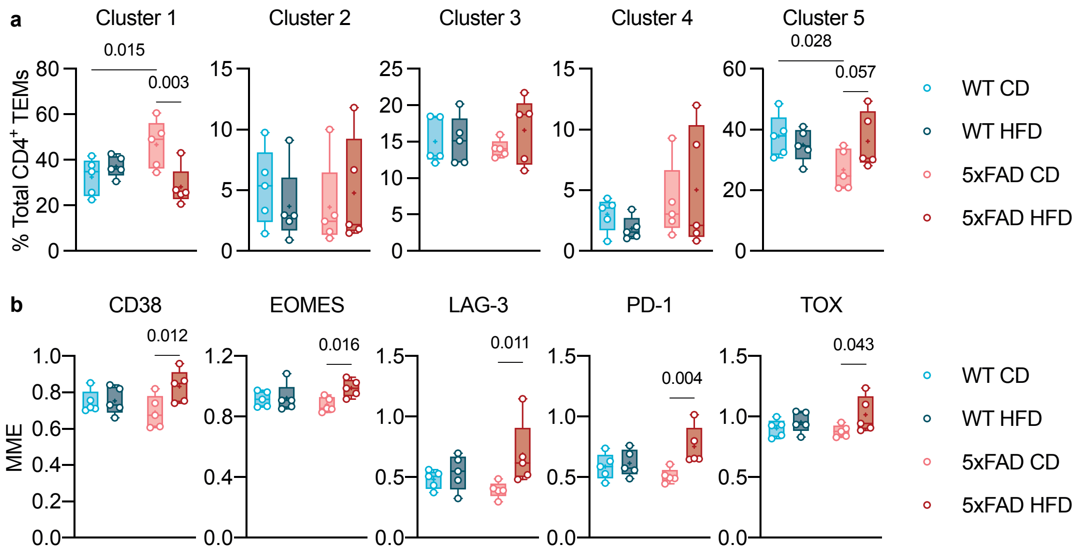

Supplementary Fig. 9. Sub-clustering analysis of the splenic CD4⁺ TEM compartment and expression of exhaustion-related markers in mice. Mice are the same described in Fig. 3e–f, sample *n*: WT CD=5, WT HFD=5, 5xFAD CD=5, 5xFAD HFD=5. **a**, Sub-clustering analysis of the CD4⁺ TEM compartment identified six clusters; plot relative to Cluster 6 is reported in Fig. 3f. **b**, HFD increased the expression of exhaustion markers in CD4⁺ TEMs in 5xFAD mice. Expression of selected exhaustion-related markers. MME, mean marker expression. **a**, **b**, Statistical analyses: two-way ANOVA followed by Fisher's LSD *post hoc* test. Box plots represent the minimum and maximum values (whiskers), the first and third quartiles (box boundaries), the median (box internal line), and the mean (cross). Source data are provided as a Source Data file.

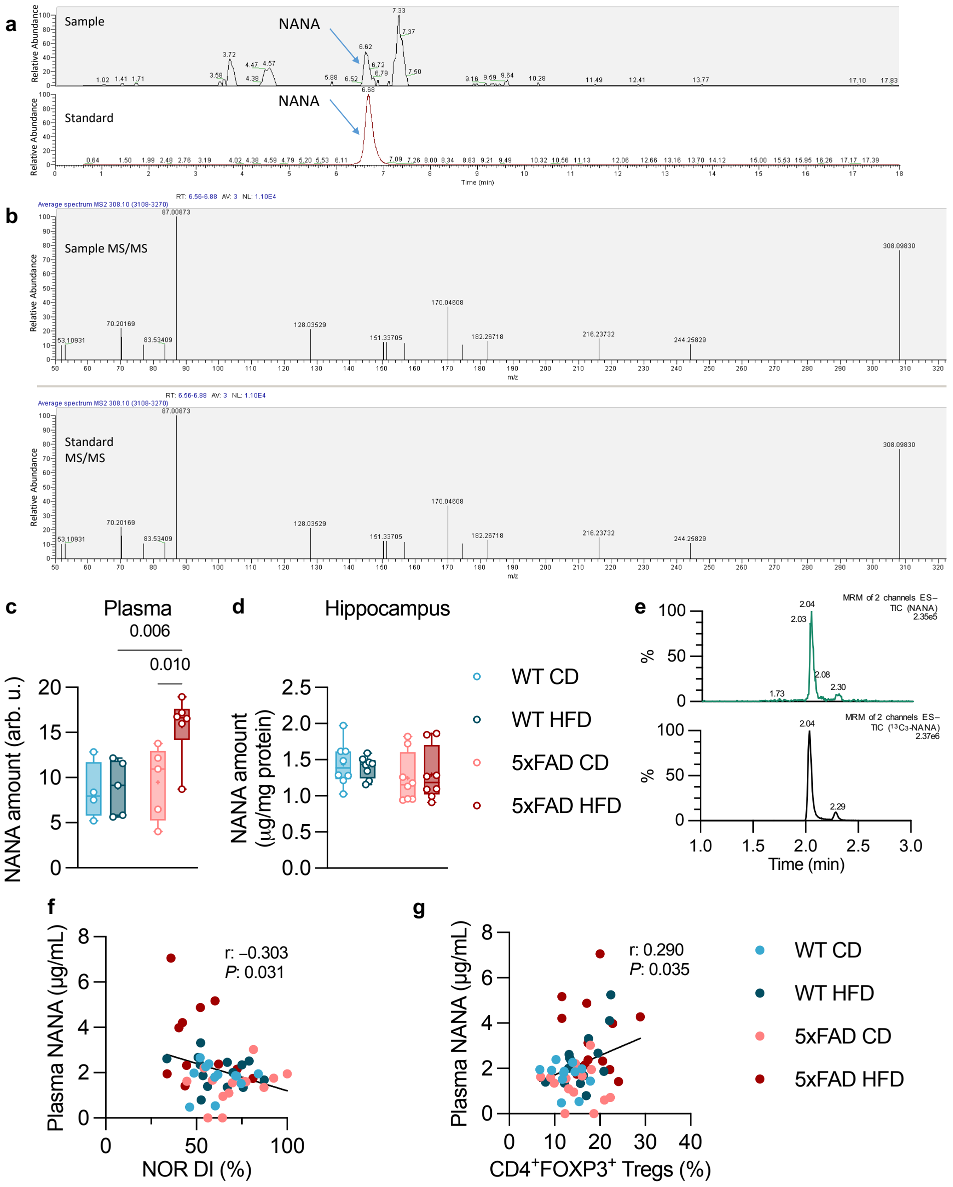

Supplementary Fig. 10: Free NANA was elevated in the plasma but comparable to controls in the hippocampus of HFD-fed 5xFAD mice. a, Representative mass chromatograms of NANA sample (top) and standard (bottom). **b**, Representative MS/MS spectra of NANA sample (top) and standard (bottom). **c**, Plasma levels of free NANA as detected by metabolite profiling; arb. u., arbitrary unit (normalized peak area/100,000). Mice are the same described in Fig. 4a–d, sample *n*: WT CD=4, WT HFD=5, 5xFAD CD=5, 5xFAD HFD=6. Statistical analysis: two-way ANOVA followed by Fisher's LSD *post hoc* test. **d**, Hippocampal levels of free NANA (TBSE-soluble fraction) as detected by LC-MS/MS. Mice from two independent experiments, sample *n*: WT CD=8, WT HFD=8, 5xFAD CD=8, 5xFAD HFD=8. Statistical analysis: two-way ANOVA

followed by Fisher's LSD *post hoc* test. **c**, **d**, Box plots represent the minimum and maximum values (whiskers), the first and third quartiles (box boundaries), the median (box internal line), and the mean (cross). **e**, Representative LC-MS/MS chromatograms of NANA standard (100 ng/mL; top) and $^{13}C_3$-NANA internal standard (1 ug/mL; bottom). **f**, **g**, Simple linear regression (black line) and Pearson's correlation (r coefficient, two-tailed *P*-value) between NANA levels, as quantified after fluorometric assay (Fig. 4e), and NOR discrimination index (DI; **f**), and between NANA levels and splenic Tregs abundance (**g**). Mice from two independent experiments, sample *n*: **f**, WT CD=12, WT HFD=14, 5xFAD CD=14, 5xFAD HFD=11; **g**, WT CD=12, WT HFD=14, 5xFAD CD=14, 5xFAD HFD=13. **c**–**g**, Source data are provided as a Source Data file.

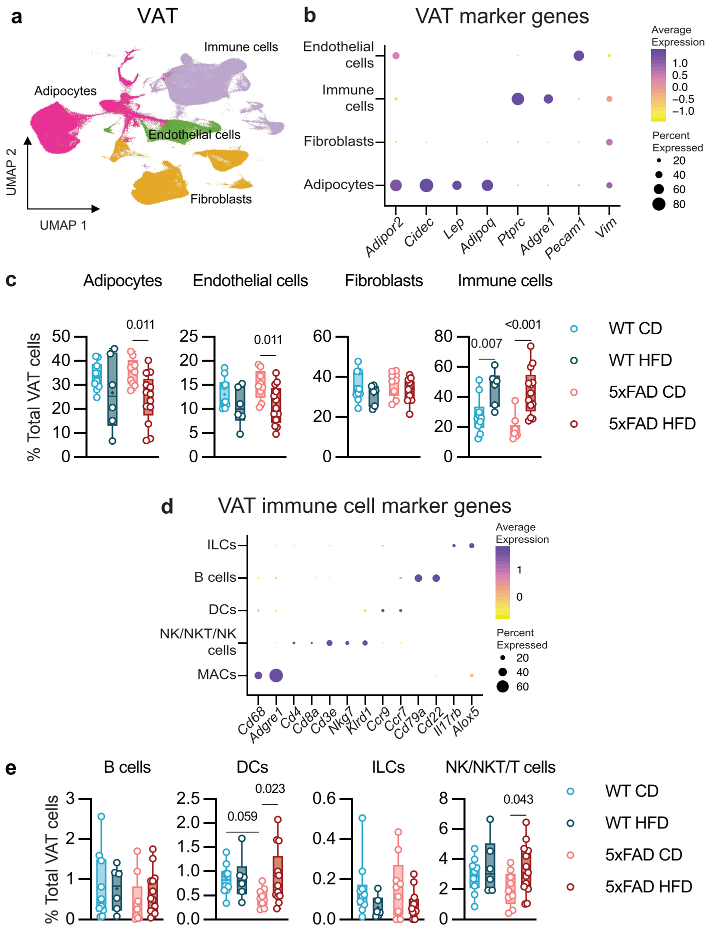

Supplementary Fig. 11: sNuc-Seq of the mouse visceral adipose tissue. Mice are the same described in Fig. 5a–d, sample *n*: WT CD=10, WT HFD=6, 5xFAD CD=9, 5xFAD HFD=13. **a**–**c**, Main cell populations in the mouse gonadal visceral adipose tissue (VAT) across all genotype and diet conditions. **a**, UMAP embedding of single nuclei profiles (sNuc-Seq), colored after *post hoc* cell type annotation. **b**, Dot plot featuring the expression of marker genes (color scale) and the percentage of cells expressing them (dot size) of VAT cell types. **c**, Changes in frequency of cell types across experimental conditions. **d**, **e**, Sub-clustering analysis of VAT immune cells. **d**, Dot plot featuring the expression of marker genes (color scale) and the percentage of cells expressing them (dot size) of VAT immune cell types. **e**, Changes in frequency of cell types across experimental conditions. Plot relative to MACs is shown in Fig. 5b. Abbreviations: DCs, dendritic cells; ILCs, innate lymphoid cells; MACs, macrophages; NK, natural killer cells; NKT, natural killer T cells. **c**, **e**, Statistical analyses: two-way ANOVA followed by Fisher's LSD *post hoc* test. Box plots represent the minimum and maximum values (whiskers), the first and third quartiles

(box boundaries), the median (box internal line), and the mean (cross). Source data are provided as a Source Data file.

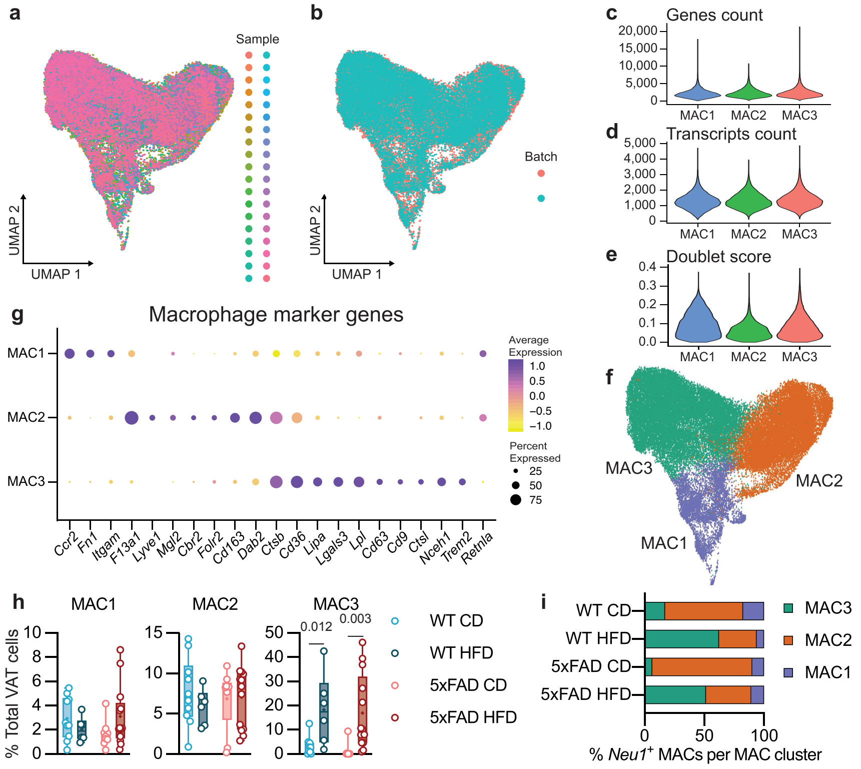

Supplementary Fig. 12: Sub-clustering analysis of mouse visceral adipose tissue macrophages. Mice are the same described in Fig. 5a–d, sample *n*: WT CD=10, WT HFD=6, 5xFAD CD=9, 5xFAD HFD=13. Three MAC clusters were identified, annotated as MAC1–3. **a**–**e**, Quality controls for visceral adipose tissue (VAT) macrophages sub-clustering analysis. **a**, **b**, Macrophage cells in VAT cluster independently of sample (**a**) and batch effects (**b**). **c**–**e**, Violin plots showing the distribution of number of genes (**c**), number of transcripts (**d**), and doublet scores (**d**) detected in each MAC cluster. **f**, VAT MAC clusters, UMAP embedding of single nuclei profiles (sNuc-Seq), colored after *post hoc* cell type annotation. **g**, Dot plot featuring the expression of marker genes (color scale) and the percentage of cells expressing them (dot size) of VAT MACs. **h**, Changes in frequency of MAC clusters across experimental conditions. Statistical analyses: two-way ANOVA followed by Fisher's LSD *post hoc* test. Box plots represent the minimum and maximum values (whiskers), the first and third quartiles (box boundaries), the median (box internal line), and the mean (cross). **i**, Stacked bar plot showing the distribution of *Neu1*-expressing macrophages in each MAC cluster across experimental conditions. Note: In one of the 5xFAD CD mice, *Neu1*-expressing macrophages were below detection level in all MAC clusters. **h**, **i**, Source data are provided as a Source Data file.

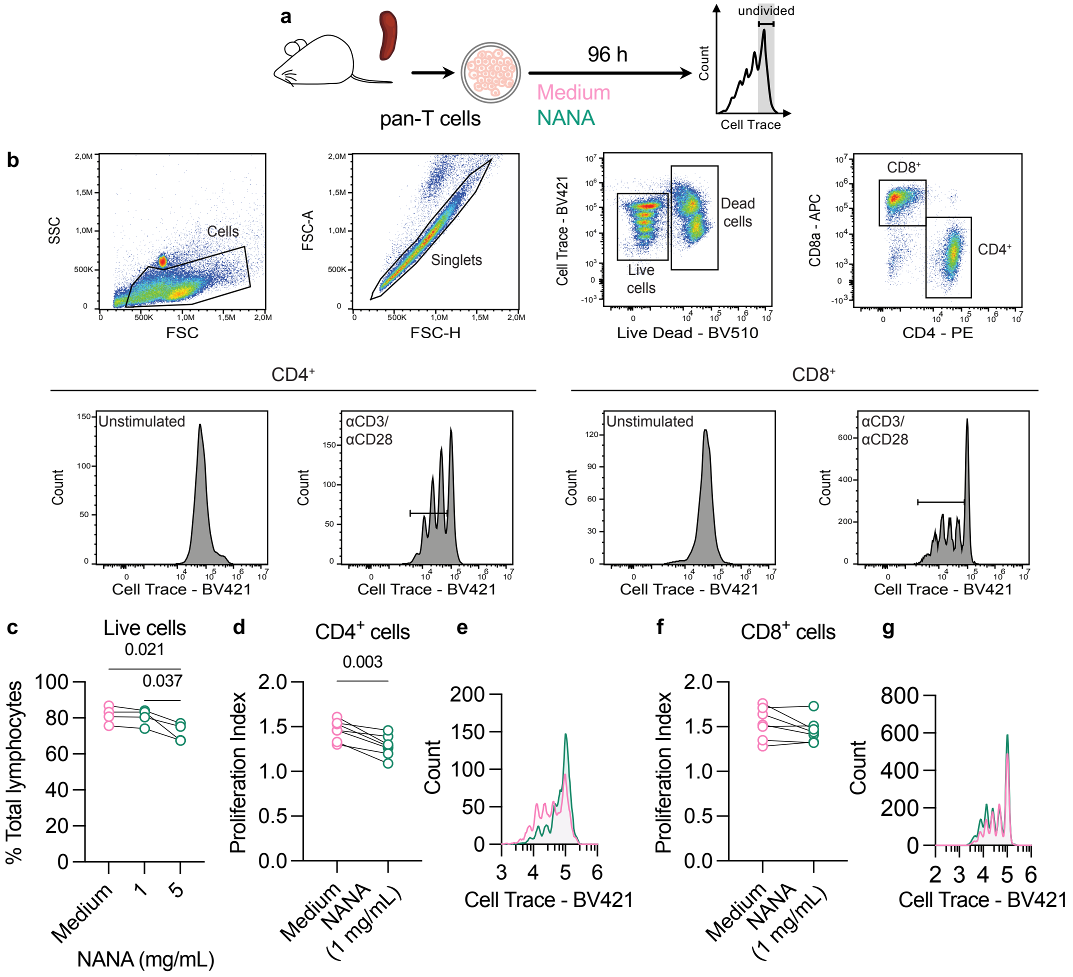

Supplementary Fig. 13: NANA treatment impaired mouse CD4⁺ T-cell proliferation *in vitro*. **a**, Schematic presentation of treatment regimen of mouse pan-T-cell cultures and proliferation assay. **b**, Flow cytometric analysis of CD4⁺ and CD8⁺ T-cell proliferation, gating strategy. **c**, Viability assay with two different concentrations of NANA. Data from one experiment, sample *n*=4 mice. From each individual mouse, one aliquot of splenocytes was treated with NANA 1 mg/mL, one with NANA 5 mg/mL, and one with medium as control. Black lines connect paired points. Statistical analyses: one-way within-subjects ANOVA followed by Fisher's LSD *post hoc* test. **d–g**, NANA exposure reduced proliferation of CD4⁺ (**d**, **e**), but not CD8⁺ T cells (**f**, **g**). **e**, **g**, Histograms of representative samples. Data from two independent experiments, of which one is the same described in **c**, sample *n*=7 mice. From each individual mouse, one aliquot of splenocytes was treated with NANA 1 mg/mL, and one with medium as control. **d**, **f**, Black lines connect

paired points. Statistical analyses: paired two-tailed Student's *t*-test. **c–g**, Source data are provided as a Source Data file.

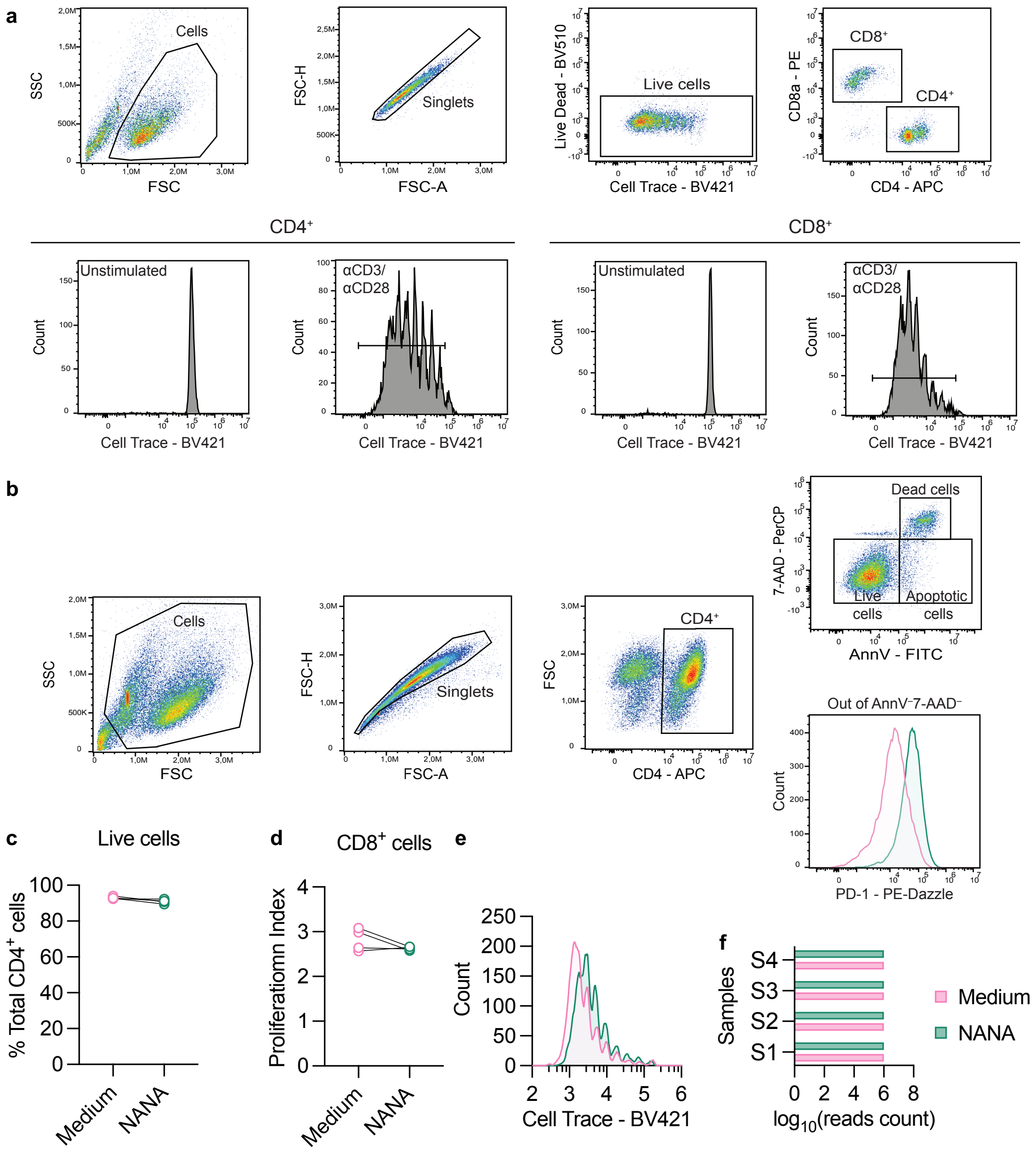

Supplementary Fig. 14: NANA treatment had no impact on human CD4⁺ T-cell viability and human CD8⁺ T-cell proliferation. **a**, **b**, Flow cytometric analysis of human CD4⁺ and CD8⁺ T-cell proliferation (**a**) and apoptosis/viability assay (**b**), gating strategy. **b**, PD-1 expression (geometric mean fluorescence intensity) was evaluated in annexin V (AnnV)/7-amino-actinomycin D (7-AAD)-double negative cells. **c**, NANA exposure did not affect CD4⁺ T-cell viability. **d**, NANA exposure did not affect human CD8⁺ T-cell proliferation. **e**, Histograms of a representative sample. **c**, **d**, Data refer to the analysis of the same samples described in Fig. 6a–e, sample *n*=4 human individuals (blood). Black lines connect paired points. Statistical analyses: paired two-tailed Student's *t*-test. **f**, Quality control for bulk RNA-Seq of human T-cell cultures.

Reads count per sample (log_{10}). For each sample, 24,594 genes were used. Data refer to the analysis of the same samples described in Fig. 6f, g, sample *n*=4 human individuals (blood). S1–4: subject 1–4. **c–f**, Source data are provided as a Source Data file.

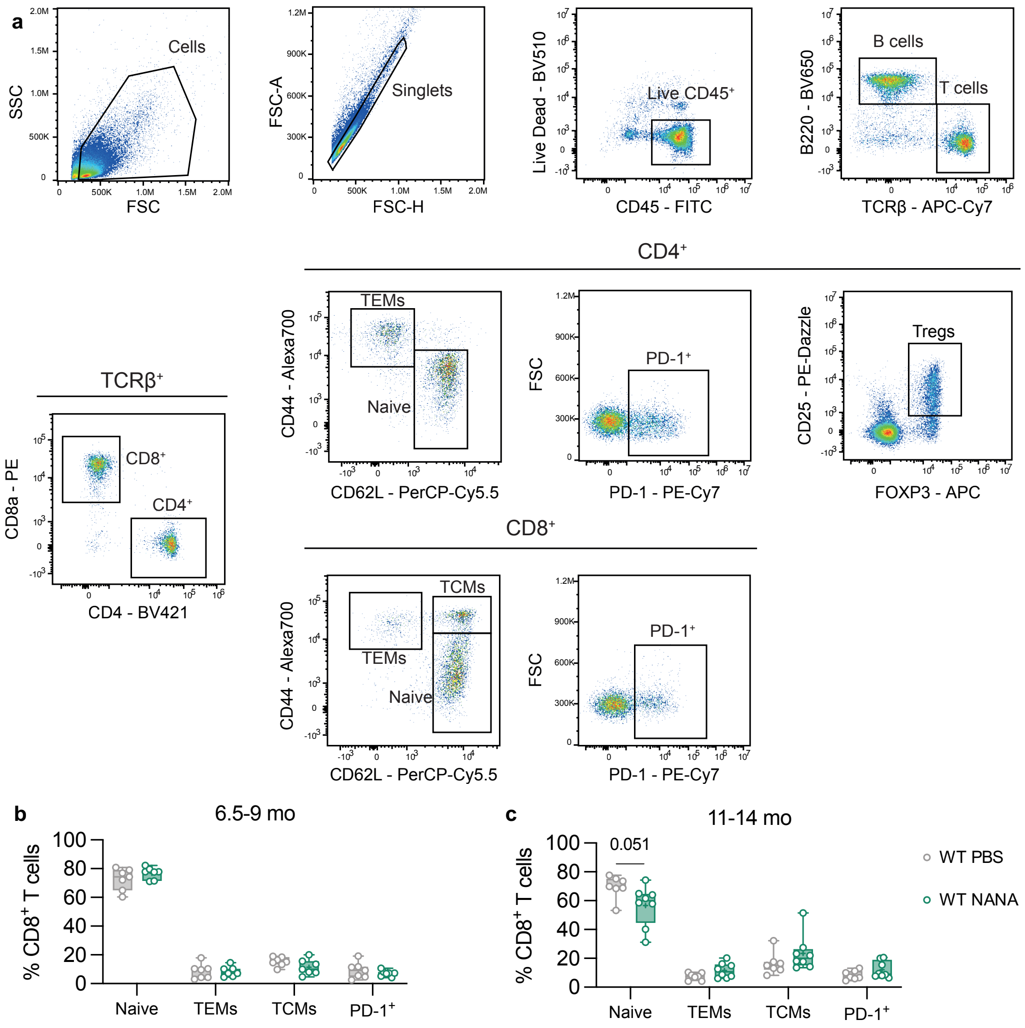

Supplementary Fig. 15: NANA treatment did not affect the splenic CD4⁺/CD8⁺ T-cell profile of young-adult mice or the splenic CD8⁺ T-cell profile of middle-aged mice. a, Flow cytometric characterization of the CD4⁺ and CD8⁺ T-cell compartments, gating strategy. **b**, **c**, Quantification of splenic frequencies of CD8⁺ naive T cells ($CD44^{low}CD62L^{high}$), CD8⁺ TEMs ($CD44^{high}CD62L^{low}$), CD8⁺ TCMs ($CD44^{high}CD62L^{high}$), and CD8⁺PD1⁺ T cells in young-adult (**b**) and middle-aged WT mice (**c**). Mice are the same described in Fig. 7b, c, sample *n*: **b**, PBS=7, NANA=7; **c**, PBS=7, NANA=8. Statistical analyses: multiple two-tailed unpaired Student's *t*-tests. Box plots represent the minimum and maximum values (whiskers), the first and third quartiles (box boundaries), the median (box internal line), and the mean (cross). Source data are provided as a Source Data file.

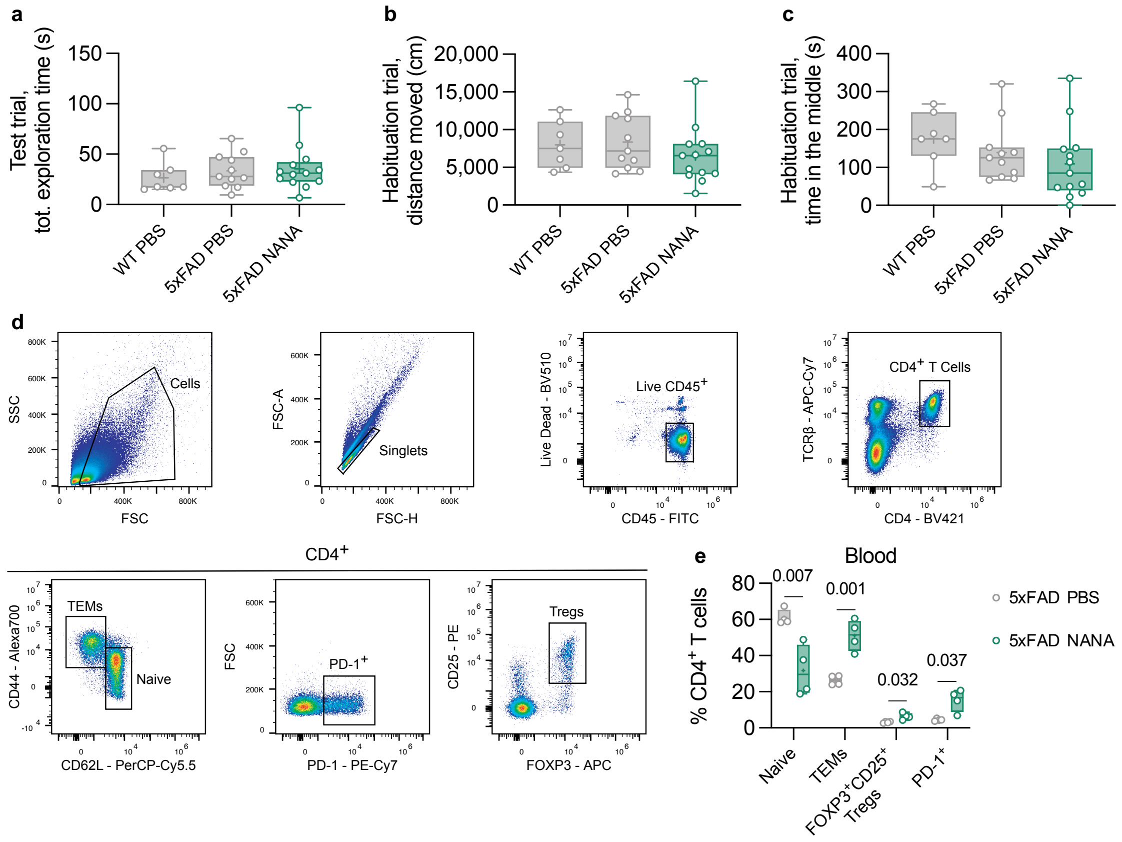

Supplementary Fig. 16: Effects of NANA administration on locomotor activity/anxiety and spleen and blood T-cell profile of 5xFAD mice 3 weeks after end of treatment. a, Total exploration time during the test trial of the NOR test. **b**, Distance moved in the empty arena during the habituation trial of the NOR test. **c**, Time spent in the middle of the empty arena during the habituation trial of the NOR test. Mice are the same described in Fig. 7e, sample *n*: WT PBS=7, 5xFAD PBS=11, 5xFAD NANA=13. Statistical analyses: one-way ANOVA followed by Fisher's LSD *post hoc* test. **d**, **e**, Flow cytometric characterization of the $CD4^+$ T-cell compartment in the blood of 5xFAD mice treated with NANA. **d**, Gating strategy. **e**, Blood $CD4^+$ T-cell profile. Mice are from one of the three cohorts described in Fig. 7e, age at cognitive assessment: 9 mo, sample *n*: 5xFAD PBS=4, 5xFAD NANA=4. Statistical analyses: multiple two-tailed unpaired Student's *t*-tests. Note: For $FOXP3^+CD25^+$ Tregs and PD-1^+, Welch's correction was used (Methods, *Statistical analyses* section). **a–c**, **e**, Box plots represent the minimum and maximum values (whiskers), the first and third quartiles (box boundaries), the median (box internal line), and the mean (cross). Source data are provided as a Source Data file.

Supplementary Table 1. Fluorochrome-labeled monochrome antibodies used in the study.

Antigen	Fluorophore	Dilution	Supplier	Cat. #
CD62L	PE-Cy7	1:150	Biolegend	104418
CD11b	PerCP-Cy5.5	1:200	Biolegend	101228
CD44	AlexaFluor700	1:150	Biolegend	103026
TCRβ	APC-Cy7	1:150	Biolegend	109220
CD4	BV421	1:150	Biolegend	100544
CD45	FITC	1:150	Biolegend	103108
FOXP3	PE	1:150	Biolegend	126404
B220	BV650	1:150	Biolegend	103241
CD8a	PE	1:150	Biolegend	100708
PD-1	PE-Cy7	1:100	Biolegend	109110
FOXP3	APC	1:100	Invitrogen	17-5773-82
CD62L	PerCP-Cy5.5	1:150	Biolegend	104432
CD4	APC	1:150	Biolegend	100412
CD4	PE	1:150	Biolegend	100408
CD8a	APC	1:200	Biolegend	100712
CD8a	PE	1:200	Biolegend	300908
CD4	APC	1:200	Biolegend	317416
PD-1	PE-Dazzle	1:100	Biolegend	329940

CD25	PE	1:100	Biolegend	162104
CD25	PE-Dazzle	1:100	Biolegend	102048

Supplementary Table 2. Heavy metal-conjugated antibodies used in the study. Anti-mouse antibodies were either purchased pre-conjugated to heavy-metal isotopes (Fluidigm) or conjugated using the MIBItag Conjugation protocol (IONpath). Fluidigm antibodies were used according to manufacturer instructions (1 µl per 3×10^6 cells). In-house conjugated antibodies were used at a dilution of 1:50.

**Antigen**	**Metal**	**Supplier**	**Cat. #**	**Clone**	**Tissue**	**In-house conj.**	**Staining step**
CD45	^{89}Y	Fluidigm	3089005B	30-F11	Sp, B		S
CD27	^{113}In	Biolegend	124202	LG.3A10	Sp	yes	S
TCRγδ	^{115}In	R&D	MAB7297	GL-3	Sp	yes	S
Ly6G	^{141}Pr	Fluidigm	3141008B	1A8	B		S
CD11c	^{142}Nd	Fluidigm	3142003B	N418	B		S
TCRβ	^{143}Nd	Fluidigm	3143010B	H57-597	Sp, B		S
B220	^{144}Nd	Fluidigm	3144011B	RA3-6B2	B		S
CD4	^{145}Nd	Fluidigm	3145002B	RM4-5	Sp, B		S, I
CD8a	^{146}Nd	Fluidigm	3146003B	53-6.7	B		S
TIGIT	^{146}Nd	Biolegend	142102	1G9	Sp	yes	S
KLRG1	^{147}Sm	R&D	MAB69441	2F1	Sp	yes	S

CD11b	^{148}Nd	Fluidigm	3148003B	M1/70	Sp, B		S
CD19	^{149}Sm	Fluidigm	3149002B	6D5	B		S
CD25	^{150}Nd	Fluidigm	3150002B	3C7	Sp		S
CD8a	^{153}Eu	Fluidigm	3153012B	53-6.7	Sp		S
CD38	^{154}Sm	Biolegend	102702	90	Sp	yes	S
CCR6	^{156}Gd	Fluidigm	3156016A	29-2L17	Sp		S
CD103	^{157}Gd	Biolegend	121402	2E7	Sp	yes	S
PD-1	^{159}Tb	Fluidigm	3159006B	RMP1-30	Sp		S
CD5	^{160}Gd	Fluidigm	3160002B	53-7.3	B		S
LEPR	^{163}Dy	R&D	AF497	Polyclonal	Sp	yes	S
Ly6AE	^{164}Dy	Fluidigm	3164005B	D7	B		S
CX3CR1	^{164}Dy	Fluidigm	3164023B	SA011F11	Sp		S
CD95	^{166}Er	R&D	AF435	Polyclonal	Sp	yes	S
CD44	^{171}Yb	Fluidigm	3171003B	IM7	Sp		S
LAG-3	^{174}Yb	Fluidigm	3174019B	C9B7W	Sp		S
CD127	^{175}Lu	Fluidigm	3175006B	3174013B	Sp		S
ICOS	^{176}Yb	Fluidigm	3176014B	7E.17G9	Sp		S
EOMES	^{152}Sm	Biolegend	MAB8889	1219A	Sp	yes	I
TOX	^{155}Gd	Thermo Fisher	14-6502-95	TXRX10	Sp	yes	I

FOXP3	^{158}Gd	Fluidigm	3158003A	FJK-16s	Sp	I
TBET	^{161}Dy	Fluidigm	3161014B	4B10	Sp	I
TNF-α	^{162}Dy	Fluidigm	3162002B	MP6-XT22	Sp	I
Ly6C	^{162}Dy	Fluidigm	3162014B	HK1.4	B	S
IFN-γ	^{165}Ho	Fluidigm	3165003B	XMG1.2	Sp	I
IL-6	^{167}Er	Fluidigm	3167003B	MP5-20F3	Sp	I
IL-17a	^{169}Tm	Fluidigm	3169005B	TC11-18H10.1	Sp	I

Abbreviations: B, blood; I, intracellular staining; S, surface staining; Sp, spleen.
