# Supplementary material for: N-acetylneuraminic acid links immune exhaustion and accelerated memory deficit in diet-induced obese Alzheimer’s disease mouse model
Source: Nat Commun. 2023 Mar 9;14:1293. doi: 10.1038/s41467-023-36759-8 (PMC9998639; doi:10.1038/s41467-023-36759-8)
Supplement: Supplementary file 2 — Description of Additional Supplementary Files [file 41467_2023_36759_MOESM2_ESM.pdf]

Description of Additional Supplementary Files

File Name: Supplementary Data 1.

Description: Gene set enrichment analysis (hypergeometric test P-values, FDR <0.050) of the dentate gyrus cluster DG1 after sNuc-Seq of the mouse hippocampus.

File Name: Supplementary Data 2.

Description: Differentially upregulated genes (MAST test P-values, FDR<0.050) in the T-cells cluster versus all other immune cells after sNuc-Seq of the mouse hippocampus.

File Name: Supplementary Data 3.

Description: Plasma metabolites identified after metabolite profiling. The statistics related to the Cell Means Model (one-way omnibus ANOVA test P-values, coefficients and Pvalues for each genotype:diet condition), Spearman correlations (ϱ coefficients and two-tailed P-values), and pairwise comparisons of each metabolite's abundance across genotype and diet groups (two-tailed unpaired Student's t-tests) are also included.

File Name: Supplementary Data 4.

Description: Differentially regulated genes (DESeq2 P-values, FDR <0.050) in human T-cell cultures after NANA treatment relative to medium-treated controls.

File Name: Supplementary Data 5.

Description: Gene set enrichment analysis (hypergeometric test P-values, FDR <0.050) of human T-cell cultures after NANA treatment relative to medium-treated controls.
