# Supplementary material for: N-acetylneuraminic acid links immune exhaustion and accelerated memory deficit in diet-induced obese Alzheimer’s disease mouse model
Source: Nat Commun. 2023 Mar 9;14:1293. doi: 10.1038/s41467-023-36759-8 (PMC9998639; doi:10.1038/s41467-023-36759-8)
Supplement: Supplementary file 9 — Reporting Summary [file 41467_2023_36759_MOESM9_ESM.pdf]

nature portfolio

Corresponding author(s): Michal Schwartz, Naomi Habib, Anna Greka, Stefano Suzzi

Last updated by author(s): Feb 3, 2023

# Reporting Summary

Nature Portfolio wishes to improve the reproducibility of the work that we publish. This form provides structure for consistency and transparency in reporting. For further information on Nature Portfolio policies, see our Editorial Policies and the Editorial Policy Checklist.

## Statistics

For all statistical analyses, confirm that the following items are present in the figure legend, table legend, main text, or Methods section.

| n/a | Confirmed | |
|---|---|---|
| ☐ | ☒ | The exact sample size (*n*) for each experimental group/condition, given as a discrete number and unit of measurement |
| ☐ | ☒ | A statement on whether measurements were taken from distinct samples or whether the same sample was measured repeatedly |
| ☐ | ☒ | The statistical test(s) used AND whether they are one- or two-sided<br>*Only common tests should be described solely by name; describe more complex techniques in the Methods section.* |
| ☒ | ☐ | A description of all covariates tested |
| ☐ | ☒ | A description of any assumptions or corrections, such as tests of normality and adjustment for multiple comparisons |
| ☐ | ☒ | A full description of the statistical parameters including central tendency (e.g. means) or other basic estimates (e.g. regression coefficient) AND variation (e.g. standard deviation) or associated estimates of uncertainty (e.g. confidence intervals) |
| ☐ | ☒ | For null hypothesis testing, the test statistic (e.g. *F*, *t*, *r*) with confidence intervals, effect sizes, degrees of freedom and *P* value noted<br>*Give P values as exact values whenever suitable.* |
| ☒ | ☐ | For Bayesian analysis, information on the choice of priors and Markov chain Monte Carlo settings |
| ☒ | ☐ | For hierarchical and complex designs, identification of the appropriate level for tests and full reporting of outcomes |
| ☐ | ☒ | Estimates of effect sizes (e.g. Cohen's *d*, Pearson's *r*), indicating how they were calculated |

*Our web collection on statistics for biologists contains articles on many of the points above.*

## Software and code

Policy information about availability of computer code

| | |
|---|---|
| Data collection | Behaviour data were recorded using the EthoVision XT 11 automated tracking system (Noldus). Flow cytometry data were acquired with CytExpert on a CytoFLEX S system (Beckman Coulter). Mass cytometry data were acquired using the the Helios™ II CyTOF® system (Fluidigm). Data from fluorometric assays, BCA assays, or ELISA were acquired using an Infinite 200 PRO or a Spark microplate readers (Tecan). Images were acquired using a fluorescence microscope (E800, Nikon) equipped with a digital camera (DXM 1200F, Nikon), and with a ×20 NA 0.50 objective lens (Plan Fluor, Nikon). Metabolite profiling data were acquired using Acquity I class UPLC System combined with mass spectrometer Q Exactive Plus Orbitrap™ (Thermo Fisher) that was operated in a negative ionisation mode; the LC separation was done using the SeQuant Zic-pHilic (150×2.1mm) with the SeQuant guard column (20×2.1mm; Merck). Acquisition of LC-MS/MS data related to hippocampal NANA were done using Masslynx and Targetlynx software (v.4.1, Waters). NANA amount in the hippocampus was measured using Acquity I-class UPLC system (Waters) and Xevo TQ-S triple quadrupole mass spectrometer (Waters). Bulk RNA-seq was done using NEBNext Ultra II directional. Nuclei were counted and imaged using the Luna-FL and Nexcelom Cellometer Vision. sNuc-seq libraries were made using 10X Genomics single-cell 3' Next GEM V3.1 assay, sequenced on Illumina HiSeqX and NextSeq500, and quality controlled using Agilent TapeStation and BioAnalyzer systems (with the provided softwares). The relevant codes used for computational analysis are available at https://github.com/naomihabiblab/HighFatDiet_in_AD. |
| Data analysis | Flow cytometry data were analysed using FlowJo v10 (Tree Star). Mass cytometry data were analysed using FlowJo, and R (R Core Team, 2017). FlowSOM k-NN clustering and two-dimensions UMAP projections were calculated using the CyTOF workflow package (v. 1.2) Microscopic images were analysed using either the Image-Pro Plus software (Media Cybernetics) or ImageJ (NIH). Polar metabolites data analysis was done using TraceFinder (Thermo Fisher). Analysis of LC-MS/MS data related to hippocampal NANA were done using MassLynx and TargetLynx software (v.4.1, Waters). RNA-seq data analysis was done using the commercial softwares DESeq2 or Cellranger (V5) and downstream analysis was done in R using code written by us (available on github), publicly available R packages for statistics, machine learning, visualizations, and vector manipulations, including packages for analysis of scRNA-seq data: Seurat (V4), DoubletFinder, CellBender |

(V2), Python's sklearn package 1.0.2. Statistical analyses were carried out using GraphPad Prism version 9.0, R, and Microsoft Excel.

For manuscripts utilizing custom algorithms or software that are central to the research but not yet described in published literature, software must be made available to editors and reviewers. We strongly encourage code deposition in a community repository (e.g. GitHub). See the Nature Portfolio guidelines for submitting code & software for further information.

## Data

Policy information about availability of data

All manuscripts must include a data availability statement. This statement should provide the following information, where applicable:

- Accession codes, unique identifiers, or web links for publicly available datasets
- A description of any restrictions on data availability
- For clinical datasets or third party data, please ensure that the statement adheres to our policy

Source data used for generation of Figures are provided as a Source Data file. The list of the metabolites identified after plasma metabolite profiling, the metabolite identification criteria, and their relative abundance are included in the Source Data file (“identified metabolites” tab). The raw and processed sequencing data generated in this study are publicly available and have been deposited in the Gene Expression Omnibus database under accession code GSE197082 [https://www.ncbi.nlm.nih.gov/geo/query/acc.cgi?acc=GSE197082]. The accession code is a SuperSeries comprising both the single-nucleus RNA-seq data (SubSeries GSE198835 [https://www.ncbi.nlm.nih.gov/geo/query/acc.cgi?acc=GSE198835]) and the bulk RNA-seq data (SubSeries GSE198144 [https://www.ncbi.nlm.nih.gov/geo/query/acc.cgi?acc=GSE198144]). For the analysis of bulk RNA-seq data of human lymphocyte cultures, fastq files were aligned to the human genome assembly hg38 [https://genome.ucsc.edu/cgi-bin/hgTracks?db=hg38&lastVirtModeType=default&lastVirtModeExtraState=&virtModeType=default&virtMode=0&nonVirtPosition=&position=chr2%3A25160915%2D25168903&hgsid=1553288579_kLzIbHUuXSroMXi12UKhdVkfiMOR]. All other raw data are available from the corresponding authors upon request.

## Human research participants

Policy information about studies involving human research participants and Sex and Gender in Research.

| | |
|---|---|
| Reporting on sex and gender | Blood from two male and two female individuals was collected. Sex was not considered in the study design. |
| Population characteristics | All subjects (average age 34 years, both sexes) were free from acute infectious diseases and in good physical condition |
| Recruitment | All donors were informed on the purpose of the study and gave their consent. Healthy volunteers were reached out directly by the authors. No self-selection bias is expected for the type of assay used. |
| Ethics oversight | The study was approved by the Institutional Review Board of the Rambam and Galilee Medical Centers (application numbers: 0013-20-RMB/66756). |

Note that full information on the approval of the study protocol must also be provided in the manuscript.

# Field-specific reporting

Please select the one below that is the best fit for your research. If you are not sure, read the appropriate sections before making your selection.

[x] Life sciences [ ] Behavioural & social sciences [ ] Ecological, evolutionary & environmental sciences

For a reference copy of the document with all sections, see nature.com/documents/nr-reporting-summary-flat.pdf

# Life sciences study design

All studies must disclose on these points even when the disclosure is negative.

| | |
|---|---|
| Sample size | No statistical method was used to predetermine sample sizes, which were chosen with adequate statistical power based on the literature and past experience (Baruch et al. Nat Med 2016; Rosenzweig et al. Nat Commun 2019; Ben-Yehuda et al. Mol Neurodegener 2021; Dvir, Castellani, Arad et al. Nat Aging 2022). The specific sample sizes and tests used to analyze each set of experiments are indicated in the Figure legends. |
| Data exclusions | For sNuc-Seq of the hippocampus, n=7 samples were originally included from each genotype and diet group, for a total of n=28 samples, which yielded 237,631 single nuclei profiles after filtering. For quantifications and statistical analyses, however, 219,237 single nuclei only were included after exclusion of two mice due to technical artifacts that hampered their annotation to a specific member of one of the experimental groups. For cognitive assessment with the NOR test, mice that were homozygotes for the Pde6brd1 allele, which causes visual impairment, were not used. For the study of the effects of NANA administration on novelty discrimination (NOR test; Fig. 7d, e and Supplementary Fig. 16a–c), four independent experiments were conducted using 9-12-mo female mice. Of these four experiments, one could not be included due to complete loss of object discrimination in the PBS-injected 5xFAD controls at the time when the animals were tested (12 mo), which could not allow to detect further exacerbation by the treatment. No animal was excluded from analyses, except those removed before experimental endpoint according to IACUC guidelines, or because of technical reasons detailed as follows: for microscopic image analysis, poorly stained or overstained sections or slides were not included; for flow and mass cytometry, samples with not enough cells to proceed with the analysis or samples in which the staining did not work were not included; for the analysis of the effects of NANA administration on the spleen immune profile of middle-aged WT mice (Fig. 7c and Supplementary Fig. 15c), one PBS-injected animal was not |

| | |
|---|---|
| | included due to its statistics across most immune cell populations (1.5x InterQuartile Range method); for the analysis of the effects of NANA administration on novelty discrimination (NOR test; Fig. 7e and Supplementary Fig. 16a–c), one PBS-injected WT mouse was not included in all tested behavioral parameters due to freezing during the test trial of the NOR assay; for the analysis of the effects of NANA administration on the spleen CD4+ T-cell profile of 5xFAD mice (Fig. 7f), one PBS-injected mouse was not included due to its statistics across all immune cell populations (1.5x InterQuartile Range method). |
| Replication | When animals from different cohorts/experiments were merged for presentation, the number of cohorts/experiments considered is indicated in the Figure legends. For comorbidity studies (Figures 1–5, Supplementary Figures 1–12), the data herein presented originated from five independent cohorts. Animals used for the mass cytometry analysis of the blood immune profile (Supplementary Fig. 6a–c) were selected from the first cohort; animals used for the sNuc-Seq of the hippocampus (Fig. 2a–m, Supplementary Figures 4 and 5) were selected from all five cohorts; animals used for the sNuc-Seq of the VAT (Fig. 5a–d, Supplementary Figures 11 and 12) were selected from the first three cohorts; animals used for all other analyses were selected from the last two cohorts. For the study on the effects of NANA administration on the spleen immune profile of young-adult and middle-aged WT mice (Fig. 7a–c and Supplementary Fig. 15a–c), four independent experiments were conducted: two with young-adult mice (6.5-9 mo), and two with middle-aged (11-14 mo) mice. For the study of the effects of NANA administration on novelty discrimination (NOR test; Fig. 7d, e and Supplementary Fig. 16a–c), four independent experiments were conducted using 9-12-mo female mice. Of these four experiments, one could not be included due to complete loss of object discrimination in the PBS-injected 5xFAD controls at the time when the animals were tested (12 mo), which could not allow to detect further exacerbation by the treatment. For in vitro studies with mouse lymphocytes, two independent experiments were conducted: one simultaneously testing two doses of NANA, 1 and 5 mg/mL (Supplementary Fig. 13c); and one testing 1 mg/mL of NANA. For in vitro studies with human lymphocytes, we performed one pilot study using 10 and 25 mg/mL of NANA. We observed that 25 mg/mL of NANA suppressed both CD4+ and CD8+ T-cell proliferation, whereas 10 mg/mL of NANA had no effect. Since the data from mouse studies in vivo (HFD-fed 5xFAD mice, Figure 3 and Supplementary Figure 7; NANA-injected mice, Fig. 7a–c and Supplementary Figure 15) and in vitro (NANA-treated pan-T-cell cultures, Supplementary Figure 13) suggested a specific impact on CD4+ T cells, with only a minor effect on CD8+ T cells, in a subsequent single experiment we used an intermediate concentration of 12.5 mg/mL of NANA (Figure 6 and Supplementary Figure 14). |
| Randomization | Animals were randomly allocated to experimental groups balancing sex and genotype. Sample selection for subsequent analyses such as CyTOF, sNuc-Seq, and metabolite profiling was based on behavioral, metabolic, flow-cytometric, and/or histological analyses. For in vitro experiments with both mouse and human lymphocytes, subjects were not divided in groups, but from each subject (mouse or human) one aliquot of cells was treated with NANA and another one with medium as control. |
| Blinding | Investigators were blind to animal identity during experiments and outcome assessment, except during behavior experiments in comorbidity studies, where diet groups, but not genotypes, were obvious. For in vitro experiments with both mouse and human lymphocytes, blinding was not relevant, as data were acquired under the same conditions using the software described in the Methods. |

# Reporting for specific materials, systems and methods

We require information from authors about some types of materials, experimental systems and methods used in many studies. Here, indicate whether each material, system or method listed is relevant to your study. If you are not sure if a list item applies to your research, read the appropriate section before selecting a response.

## Materials & experimental systems

| n/a | Involved in the study |
|---|---|
| ☐ | ☒ Antibodies |
| ☒ | ☐ Eukaryotic cell lines |
| ☒ | ☐ Palaeontology and archaeology |
| ☐ | ☒ Animals and other organisms |
| ☒ | ☐ Clinical data |
| ☒ | ☐ Dual use research of concern |

## Methods

| n/a | Involved in the study |
|---|---|
| ☒ | ☐ ChIP-seq |
| ☐ | ☒ Flow cytometry |
| ☒ | ☐ MRI-based neuroimaging |

## Antibodies

| | |
|---|---|
| Antibodies used | For immunohisdtochemistry, the following primary antibodies were used: mouse anti-human Aβ (1:150; Covance); chicken anti-GFAP (1:150; Abcam). Cy2/Cy3-conjugated anti-mouse/chicken secondary antibodies (1:150; Jackson Immunoresearch) were used. For flow cytometry, the fluorochrome-labelled antibodies and the dilutions used in the study are listed in Supplementary Table 1. For mass cytometry, the heavy-metal-conjugated antibodies, the clone names of the monoclonal antibodies, and the dilutions used in the study are listed in Supplementary Table 2. |
| Validation | For flow cytometry experiments, antibodies were used according to manufacturer's instructions and each antibody was further compared (separately) to an unstained sample or fluorescence-minus-one controls. For intracellular staining of cytokines after phorbol 12-myristate 13-acetate/ionomycin stimulation, non-stimulated control was included. For immunostaining, each immunofluorescence staining was preliminarily calibrated by comparing stained samples to a sample that was incubated with secondary antibody only. |

# Animals and other research organisms

Policy information about studies involving animals; ARRIVE guidelines recommended for reporting animal research, and Sex and Gender in Research

| | |
|---|---|
| Laboratory animals | Female and male mice were bred and maintained by the Animal Breeding Centre of the Weizmann Institute of Science. Housing conditions were: 12-hour dark/light cycle (lights on at 8 am), temperature 22°C, humidity 30-70%. For comorbidity studies, heterozygous 5xFAD transgenic mice (line Tg6799, The Jackson Laboratory) on a C57/BL6-SJL background and age-matched wild-type (WT) controls were used. The diet regimen was administered from 2 to 9 months of age. For the study of the effects of NANA on the immune system, female and male WT mice on the C57/BL6-SJL or C57/BL6 strains were used (6-7 and 11-14 months of age). For the study of the effects of NANA on novelty discrimination, female C57/BL6-SJL 5xFAD and age-matched WT controls were used (9-12 months of age). Mice allocated for behavioral studies or NANA/PBS injections were switched to a 24 h reversed dark-light cycle (lights on from 8 pm to 8 am) at least 7 days prior to behavior assessment, and maintained in the regimen until experimental endpoint. |
| Wild animals | The study did not involve wild animals. |
| Reporting on sex | Mice of both sexes were included in all experiments, except in the following cases: only male mice were used for plasma metabolite profiling (Fig. 4a–d and Supplementary Fig. 10a–c); only female mice were used for cognitive assessment and immune profiling following NANA/PBS administration to 5xFAD mice and age-matched WT controls (Fig. 7d–f and Supplementary Fig. 16a–e). |
| Field-collected samples | The study did not involve data collected from the field. |
| Ethics oversight | All experiments detailed herein complied with the regulations formulated by the Institutional Animal Care and Use Committee (IACUC) of the Weizmann Institute of Science (application numbers: 03960618-3, 01200121-2, 03230322-2). |

Note that full information on the approval of the study protocol must also be provided in the manuscript.

# Flow Cytometry

## Plots

Confirm that:

- [x] The axis labels state the marker and fluorochrome used (e.g. CD4-FITC).
- [x] The axis scales are clearly visible. Include numbers along axes only for bottom left plot of group (a 'group' is an analysis of identical markers).
- [x] All plots are contour plots with outliers or pseudocolor plots.
- [x] A numerical value for number of cells or percentage (with statistics) is provided.

## Methodology

| | |
|---|---|
| Sample preparation | Spleens were mashed with the plunger of a syringe and treated with ACK (ammonium-chloride-potassium) lysis buffer (Gibco™) to remove erythrocytes. Splenocytes were filtered through a 70 µm nylon mesh and used fresh. |
| Instrument | CytoFLEX (Beckman Coulter) |
| Software | CytExpert, FlowJo v10 (Tree Star) |
| Cell population abundance | Cell population abundance is reported in the relevant bar plots as percentage of parent population. |
| Gating strategy | Cells were gated using FSC/SSC plot and singlets gated using FSC-A/FSC-H plot. For the characterization of CD4+/CD4- compartments in mice used for comorbidity studies, CD11b-/TCRb+ T cells were gated from CD45+ cells, and further gated according to the experimental design (CD4+CD44lowCD62Lhigh naive cells, CD4+CD44highCD62Llow TEMs, CD4+FOXP3+ Tregs, CD4-CD44lowCD62Lhigh naive cells, CD4-CD44highCD62Llow TEMs, CD4-CD44highCD62Lhigh TCMs). For the characterization of the spleen CD4+/CD8+ compartments in young-adult and middle-aged WT mice treated with NANA/PBS, live CD45+ cells were gated after live/dead staining and further gated for B220-TCRb+ T cells, after which CD4+ and CD8+ populations were identified and characterized according to the experimental design (CD4+CD44lowCD62Lhigh naive cells, CD4+CD44highCD62Llow TEMs, CD4+FOXP3+CD25+ Tregs, CD8+CD44lowCD62Lhigh naive cells, CD4+PD-1+ cells, CD8 +CD44highCD62Llow TEMs, CD8+CD44highCD62Lhigh TCMs, CD8+PD-1+ cells). For the characterization of the spleen and blood CD4+ T-cell compartment of 5xFAD mice trreated with NANA/PBS, live CD45+ cells were gated after live/dead staining and further gated for TCRb+CD4+ T cells, after which populations were identified and characterized according to the experimental design (CD4+CD44lowCD62Lhigh naive cells, CD4+CD44highCD62Llow TEMs, CD4+FOXP3+CD25+ Tregs, CD4 +PD-1+ cells). For the analysis of the effects of NANA on mouse pan-T-cell proliferation, live cells were gated after live/dead and Cell Trace staining, then gated for CD4 and CD8 expression. For the analysis of the effects of NANA on human pan-T-cell proliferation, live cells were gated after live/dead and Cell Trace staining, then gated for CD4 and CD8 expression. For the analysis of the effects of NANA on the expression of PD-1 in human pan-T cells, CD4+ cells were gated for live cells after AnnV/-7AAD staining, then PD-1 expression was evaluated using geometric mean fluorescent intensity. |

- [x] Tick this box to confirm that a figure exemplifying the gating strategy is provided in the Supplementary Information.
